# Intestinal Commitment and Maturation of Human Pluripotent Stem Cells Is Independent of Exogenous FGF4 and R-spondin1

**DOI:** 10.1371/journal.pone.0134551

**Published:** 2015-07-31

**Authors:** Kaisa Tamminen, Diego Balboa, Sanna Toivonen, Mikko P. Pakarinen, Zoltan Wiener, Kari Alitalo, Timo Otonkoski

**Affiliations:** 1 Research Programs Unit, Molecular Neurology and Biomedicum Stem Cell Centre, University of Helsinki, Helsinki, Finland; 2 Children’s Hospital, University of Helsinki and Helsinki University Central Hospital, Helsinki, Finland; 3 Translational Cancer Biology Program and Wihuri Research Institute, Biomedicum Helsinki, Helsinki, Finland; University Claude Bernard Lyon 1, FRANCE

## Abstract

Wnt/beta-catenin signaling plays a central role in guiding the differentiation of the posterior parts of the primitive gut tube into intestinal structures *in vivo* and some studies suggest that FGF4 is another crucial factor for intestinal development. The aim of this study was to define the effects of Wnt and FGF4 on intestinal commitment *in vitro* by establishing conditions for differentiation of human pluripotent stem cells (hPSC) into posterior endoderm (hindgut) and further to self-renewing intestinal-like organoids. The most prominent induction of the well-established intestinal marker gene *CDX2* was achieved when hPSC-derived definitive endoderm cells were treated with Wnt agonist molecule CHIR99021 during differentiation to hindgut. FGF4 was found to be dispensable during intestinal commitment, but it had an early role in repressing development towards the hepatic lineage. When hindgut stage cells were further cultured in 3D, they formed self-renewing organoid structures containing all major intestinal cell types even without exogenous R-spondin1 (RSPO1), a crucial factor for the culture of epithelial organoids derived from adult intestine. This may be explained by the presence of a mesenchymal compartment in the hPSC-derived organoids. Addition of WNT3A increased the expression of the Paneth cell marker Lysozyme in hPSC-derived organoid cultures, whereas FGF4 inhibited both the formation and maturation of intestinal-like organoids. Similar hindgut and organoid cultures were established from human induced pluripotent stem cells, implying that this approach can be used to create patient-specific intestinal tissue models for disease modeling *in vitro*.

## Introduction

During early development of the mammalian embryo, the three germ layers, ectoderm, endoderm and mesoderm, are formed upon gastrulation. In humans, the development of internal organs from the endoderm begins during the third week of gestation. The endoderm becomes patterned by molecular cues from the microenvironment, such as retinoid acid (RA) [1], fibroblast growth factors (FGFs) [2] and Wnt ligands [3]. The crosstalk between the endoderm and mesoderm has a major role in guiding the formation of the gut-tube structure [4], which becomes further patterned by the expression of different fate-specifying transcription factors, such as *Sox2* and *Hhex* in the anterior endoderm (foregut) and *Cdx2* in the posterior endoderm (hindgut). The posterior endoderm will eventually give rise to the small and large intestine.

Several studies have described successful methods for the differentiation of human pluripotent stem cells (hPSC) into definitive endoderm (DE) [5–7] and foregut derivatives such as the liver [8, 9] or pancreas [10–12]. Only few studies have reported attempts to differentiate human pluripotent stem cells into intestinal direction [13–17]. High concentration of WNT3A together with FGF4 induced hindgut development from hESC-derived endoderm, characterized by the expression of *the Caudal-related homeobox CDX2* and leading to the formation of hindgut spheroids consisting of developing epithelium surrounded by mesenchyme [17]. The synergistic action of FGF4 and WNT3A was found to be essential for hindgut specification [17]. In another study, Wnt signaling activation by GSK3β inhibitor XV was used to activate small and large intestinal gene signatures in mouse and human PSC-derived definitive endoderm [3].

The biology of adult intestinal epithelium has been extensively studied. The intestinal multipotent stem cells reside at the bottom of the epithelial crypts interspersed with Paneth cells and express *leucine-rich repeat containing G protein-coupled receptor 5 (LGR5)* [18]. The Paneth cells together with adjacent mesenchymal cells establish the proper intestinal stem cell niche partly by secreting Wnts [19]. Small intestinal epithelium forms crypt-villus structures *in vitro* in 3D-matrix [20]. These so-called organoids are dependent on the Lgr5-ligand R-spondin1 (RSPO1), which acts as an agonist of Wnt signaling [19, 21]. Wnt signaling is needed for the homeostasis of the normal intestinal epithelium and redundancy between Wnt signals from different sources has been described, since addition of Wnt ligands allowed organoid culture without Paneth cells [22].

In the pioneering study by Spence et. al (2011), hindgut stage spheroids derived from human ES cells were cultured in similar 3D conditions as used for mouse small intestinal organoids. The spheroids developed further to form organoids containing all the four major cell types found in the adult intestinal epithelium (enterocytes, Paneth cells, goblet cells and enteroendocrine cells) [17]. In contrast to adult human intestinal organoids [19, 23], the hESC-derived organoids contained also mesenchymal cells [17]. More recently, these organoids were shown to undergo significant maturation after engraftment in immunodeficient mice [24].

In another recent study, intestinal organogenesis was observed within hPSC-derived teratomas and organoid cultures were established from sorted LGR5+ cells [25]. However, these organoids had a cystic morphology, similar to organoids derived from either human embryonic intestine [26] or from adult colon [27].

In the present study, we have further investigated the process of intestinal differentiation from hPSCs, focusing first in determining the importance of FGF4 and Wnt on the initial intestinal commitment at hindgut stage, and then in testing the effects of different culture conditions on the maturation of 3D-organoids. We show that effective hindgut commitment can be obtained with GSK3β inhibitor without the WNT3A ligand. Furthermore, FGF4 is dispensable in intestinal differentiation of hPSC and inhibits the formation and maturation of intestinal-like organoid-structures. However, FGF4 has an early role in repressing differentiation towards hepatic lineage. Finally, we show that, unlike the adult intestine-derived organoids, hPSC-derived hindgut progenitors can mature to organoids containing intestinal cell types, even without exogenous Wnt signaling agonists, like R-spondin1.

## Materials and methods

### Cell lines

Human embryonic stem cell (hESC) line H9 [28] was used in all experiments for the optimization of the differentiation protocols. Human iPS cell (hiPSC) line HEL11.4, retrovirally generated from the fibroblasts of a healthy 83-year-old male [29], was used for validation of the results (S6 Fig).

### Stem cell culture and differentiation

The undifferentiated cells were cultured on Matrigel (BD Biosciences) coated plates in StemPro medium (Invitrogen) and passaged with 1 x Collagenase IV (Invitrogen). The differentiation experiments were initiated at 80% confluency. DE differentiation was started by replacing StemPro medium with RPMI 1640 (Invitrogen) supplemented with 2% B27 (Invitrogen), 1mM sodium butyrate (NaB), 75 ng/ml WNT3A and 100 ng/ml Activin A (ActA). WNT3A was concentrated with Amicon Ultra-15 Centrifugal Filter Units according to the manufacturer’s instructions (Millipore Merck KGaA, Darmstadt, Germany) from conditioned medium (CM) produced as described elsewhere [30]. The following day the medium was changed to general DE medium where the concentration of NaB was reduced to 0.5 mM. Differentiation to DE was continued for 4 days and medium was changed every day.

The differentiation was continued with hindgut induction for 4 days in DMEM/F12 containing 2% defined FBS (HyClone) with varying concentrations of rhFGF4 (R&D Systems), rhWNT3A (R&D Systems) and CHIR99021 (Stemgent), depending on the combinations tested in each experiment.

For 3D organoid formation, about 2.5x10^5^ cells were collected at day 9 and seeded into 4x50 μl drops of Matrigel. Medium was DMEM/F12 with 1% B27 (Invitrogen), supplemented with 1% N2, 1mM N-Acetylcysteine, 100ng/ml Noggin, 50 ng/ml EGF (Invitrogen), and depending on tested conditions 500 ng/ml R-spondin1, 500 ng/ml rhFGF4 (R&D Systems) or 100ng/ml WNT3A was added. Medium was changed every 4 days and organoids were passaged mechanically every 7–10 days. Organoid cultures were analyzed after 33 days in Matrigel (d42 from hPSC), unless otherwise indicated.

### Production of recombinant proteins

Human noggin and R-spondin1 recombinant proteins were produced locally. *NOG* (NP_005441) and *RSPO1* (NP_001033722.1) cDNA sequences were modified to incorporate suitable restriction sites for cloning, synthetized (GenScript, Hong Kong) and subcloned into pEFIRES-Fc vector to generate Fc-tagged constructs. Protein production in chinese hamster ovary (CHO) cells and purification was done as described elsewhere [31].

### Immunocytochemistry

Immunocytochemical staining and analysis of cells at day 5 and day 9 was performed as described earlier [7]. Organoids for immunohistochemistry were collected manually, fixed with 4% PFA and centrifuged in liquid agar. Pellets containing organoids and agar were embedded in paraffin, processed and sectioned with routine methods. Sections were deparaffinized, rehydrated and treated with 1mM EDTA buffer (pH 8) in a microwave oven to reveal antigenic sites. Sections were incubated with UltraVision Protein Block (Thermo Scientific) for 10 min in RT before overnight incubation at +4°C with the desired primary antibodies (S1 Table). The following day, sections were incubated for 30 min with appropriate secondary AlexaFluor antibodies (Invitrogen) (S2 Table.). Vectashield with DAPI (Vector Laboratories) was used for the nuclear staining of sections. The images were acquired and analyzed by using a Zeiss Axioplan 2 microscope and Axiovision software.

### Whole mount immunocytochemistry of organoids

Organoids were grown in 4-well chamber slides for whole-mount staining. The chambers were washed with PBS, fixed with 4% PFA and incubated for 1 h at RT with blocking/permeabilizing buffer (5% non-immunogenic donkey serum, 0.2% BSA, 0.3% Triton X100 in PBS). Primary antibodies for E-CAD, LYZ or VIM (S1 Table) were diluted in blocking/permeabilizing buffer and incubation was done at +4°C overnight. The following day, the chambers were washed several times with 0.3% Triton/PBS at RT. Secondary antibodies were diluted 1:500 in blocking/ permeabilizing buffer and incubation was done at +4°C overnight. On the last day, the chambers were washed with 0.3% Triton/PBS several times at RT and nuclear staining was performed by using vectashield with DAPI (Vector Laboratories). Images were acquired with Zeiss LSM 5 Duo confocal microscope. Z-stacks of images were combined using ImageJ (NIH) software. The numbers of LYZ- and VIM-positive organoids were counted manually.

### Flow cytometry

Flow cytometry analysis for the cell surface marker CXCR4 was carried out at day 5 as described earlier [7] using PE-conjugated antibodies listed in S3 Table. At day 9 the cells required a somewhat longer incubation time with TrypLE (4 min) for dissociation. FACS buffer (5% fetal calf serum (Promocell, Heidelberg, Germany) in PBS) with 1% Saponin (Fluka) was used to permeabilize the cells for day 5 SOX17 and day 9 CDX2 nuclear stainings. Primary antibodies (SOX17 R&D Systems; CDX2, Biogenex) were diluted 5 μl per 1 million cells and incubation was done at RT for 3 hours. Incubation with secondary AlexaFluor antibodies (S2 Table) was done on ice 30 min. Analysis with Becton-Dickinson FACS-Calibur and CellQuestPro-software was carried out in FACS-buffer without Saponin.

### Quantitative PCR analysis

RNA isolation, reverse transcription to cDNA and qPCR analysis were carried out as described earlier [7]. 5x HOT FIREPol EvaGreen qPCR Mix Plus (Solis BioDyne) was used and each 20 μl multiplication reaction contained 4 μl of it, 5 μl mix of F/R primers (both 2 μM in mix), 1 μl cDNA template and 10 μl of PCR grade water. The following PCR program was used: 15 min enzyme activation step at 95°C, followed by 40 cycles of 95°C, 25 s; 56°C, 25 s; 72°C, 25 s, followed by a melting step. Corbett CAS-1200 liquid handling system was used to prepare the reactions and qPCR was performed using Corbett Rotor-Gene 6000 (Corbett Life Science, Sydney, Australia). Primers are listed in S4 Table.

We analyzed the data with ΔΔCt method [32] using Cyclophilin G as the internal housekeeping gene and exogenous standard cDNA as calibrator. Fold changes were calculated relative to averaged day 0 undifferentiated cells expression levels. For H9 cells, all results were calculated from at least 3 independent experiments, except for S4 Fig. Statistical testing was done using SPSS and one-way ANOVA comparison between groups (post hoc: Tukey, Significance: *<0,05 **<0,01 and *** <0,001).

### Ethics Statement

RNA from human intestinal epithelium was isolated from intestinal biopsies taken in endoscopy from nonaffected duodenum, ileum and transverse colon of 6–8 year old Hirschprung’s disease patients during their follow-up visits. Donors of tissue samples and their guardians had provided a written consent (Helsinki and Uusimaa Hospital District decision no. 296/13/03/03/2012) for the use of the intestinal biopsy samples to generate the gene expression data for this study. The generation of the hiPSC lines used in this study was approved by the Coordinating Ethics Committee of the Helsinki and Uusimaa Hospital District (Nro 423/13/03/00/08).

## Results

### Differentiation to definitive endoderm (DE)

**Fig 1:**
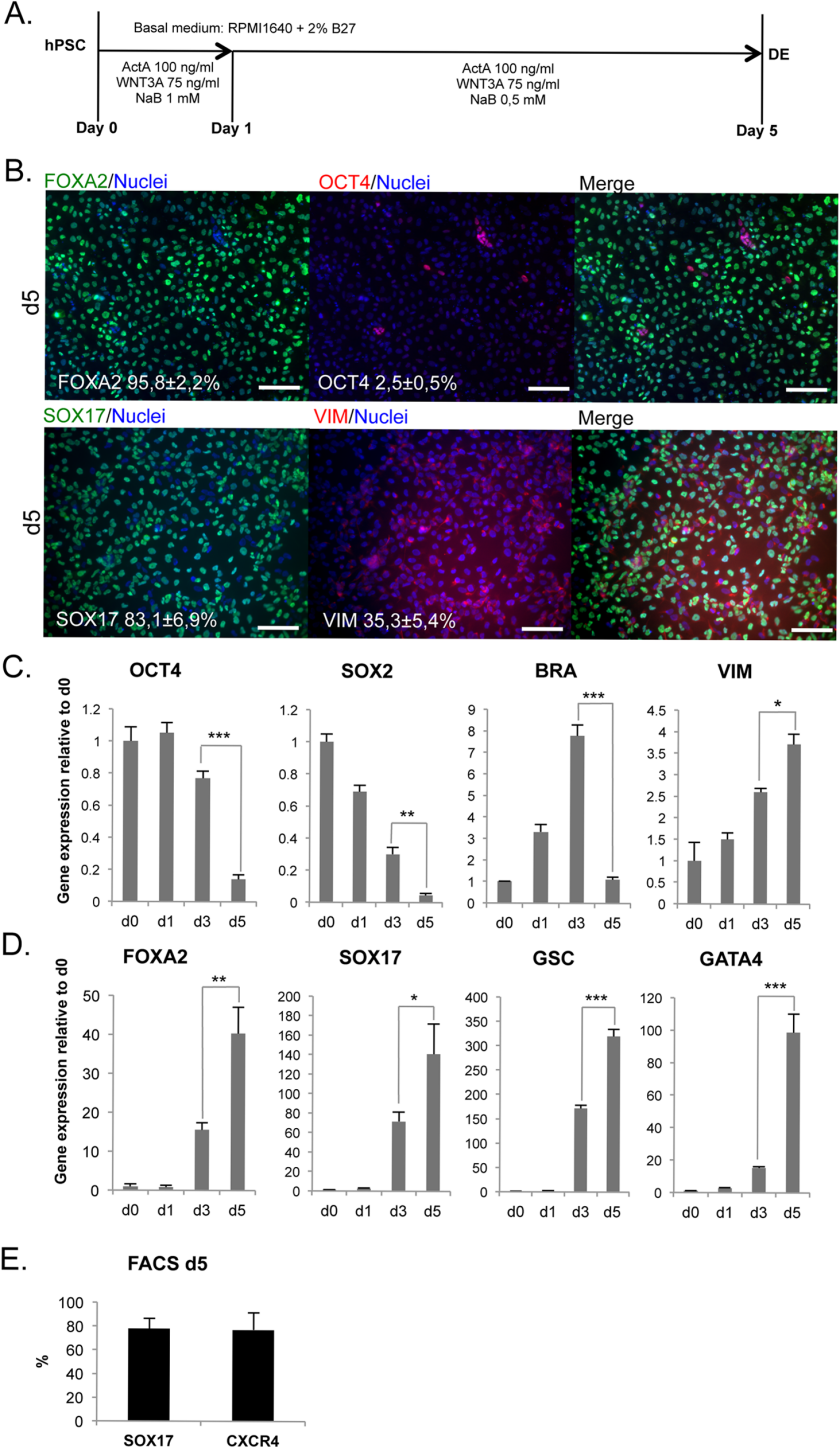
Induction of definitive endoderm (DE). A. Protocol for definitive endoderm differentiation. ActA = Activin A; NaB = Sodium butyrate. B. Immunocytochemistry for FOXA2, OCT4, SOX17 and VIM at day 5 (Scale bars 100 μm). C. Gene expression of ES-cell markers *OCT4*, *SOX2*; mesendodermal marker *BRA*; and mesenchymal *VIM* (mean ± SEM; n = 4). D. Upregulation of *FOXA2*, *SOX17*, *GSC* and *GATA4* during differentiation (mean ± SEM; n = 4). E. Cytometry analysis of DE-specific marker SOX17 (n = 5) and endodermal cell surface marker CXCR4 (n = 11) for day 5 cells (mean ± SD).

H9 cells were differentiated using a previously optimized 5-day protocol employing Activin A, WNT3A and sodium butyrate (Fig 1A). After 5 days of differentiation, the cells had a morphology characteristic of DE-cells (S1 Fig) and quantitative analysis of immunocytochemistry revealed that ≈96% of cells became positive for FOXA2 and ≈83% were positive for the more specific DE marker SOX17 (Fig 1B). At day 5, only ≈2.5% of cells were still positive for OCT4 (Fig 1B), a marker of pluripotent stem cells. Importantly, cultures contained also a significant mesenchymal VIMENTIN (VIM) positive population (≈35%) (Fig 1B) (S1 Fig). VIM and SOX17 immunoreactivities were localized mainly in separate cells, but also weakly double positive cells were identified (Fig 1B). mRNA levels of the stem cell markers *OCT4* and *SOX2* decreased strongly during days 3–5 (Fig 1C). Mesendodermal gene *BRACHUYRY* (*BRA*) was transiently upregulated at day 3, suggesting that the cells go through a mesendodermal phase. In contrast, the level of *VIMENTIN* mRNA increased steadily (Fig 1C). The mRNA levels of endodermal genes *SOX17*, *FOXA2*, *GSC*, *GATA4* were strongly upregulated during days 3–5 (Fig 1D). Flow cytometry analysis confirmed that 78±8% of the day 5 cell population was SOX17+ and 77±14% had cell surface marker CXCR4 (Fig 1E). CXCR4 is widely accepted as a specific cell surface marker of definitive endoderm cells in pluripotent stem cell differentiation [5, 33].

### Treatment with CHIR99021 efficiently induces *CDX2* expression

**Fig 2:**
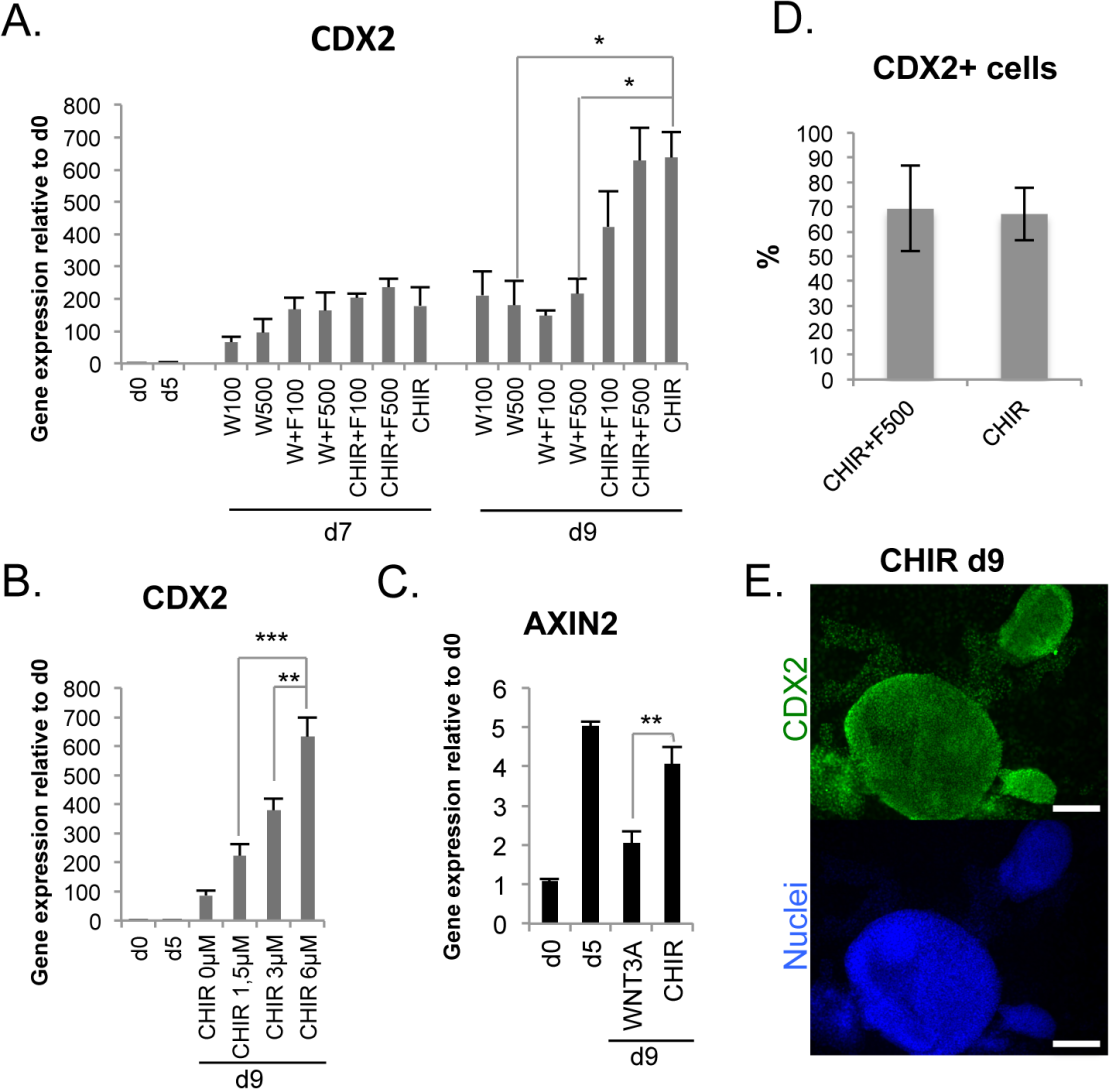
Effective hindgut induction by CHIR. A. Upregulation of *CDX2* mRNA during differentiation driven by administration of WNT3A (W), FGF4 (F) and CHIR99021 (CHIR) (mean ± SEM; n = 4–17). Numbers indicate the concentrations used in ng/ml, CHIR concentration was 3 μM. B. Dose-dependent upregulation of *CDX2* by varying concentrations of CHIR (mean ± SEM; n = 3). C. Expression of the Wnt target gene *AXIN2* at d9 after differentiation with WNT3A (500 ng/ml) or CHIR (3 μM) (mean ± SEM; n = 4). D. Cytometry analysis of CDX2+ population in day 9 cultures differentiated using either CHIR or CHIR+F500 (mean ± SD). E. Immunocytochemistry for CDX2 at day 9 after CHIR treatment (scale bars 200 μm).

We tested different combinations of WNT3A, FGF4 and CHIR99021 (CHIR) for hindgut induction using the day 5 DE-cells. CHIR activates canonical Wnt signaling pathway by inhibiting GSK3β and blocking the formation of the β-catenin destruction complex. WNT3A alone or in combination with FGF4 induced upregulation of *CDX2* expression, but when CHIR was used instead of WNT3A, *CDX2* levels were significantly increased (Fig 2A), peaking at day 9. This effect was dose-dependent (Fig 2B). CHIR treatment maintained mRNA levels of the Wnt target gene *AXIN2* upregulated to the same level as in day 5 DE-cells, whereas in WNT3A-treated hindgut stage cells *AXIN2* expression dropped (Fig 2C). At this stage the cultures became more 3-dimensional compared to earlier stages, especially in the presence of CHIR (Fig 2E) (S2 Fig). Addition of FGF4 did not modify the *CDX2* expression levels induced by WNT3A or CHIR (Fig 2A) or the proportion of CDX2 positive cells (67–69%, Fig 2D). Therefore, exogenous FGF4 could be considered as dispensable in intestinal commitment.

### FGF4 represses the hepatic differentiation lineage at hindgut stage

**Fig 3:**
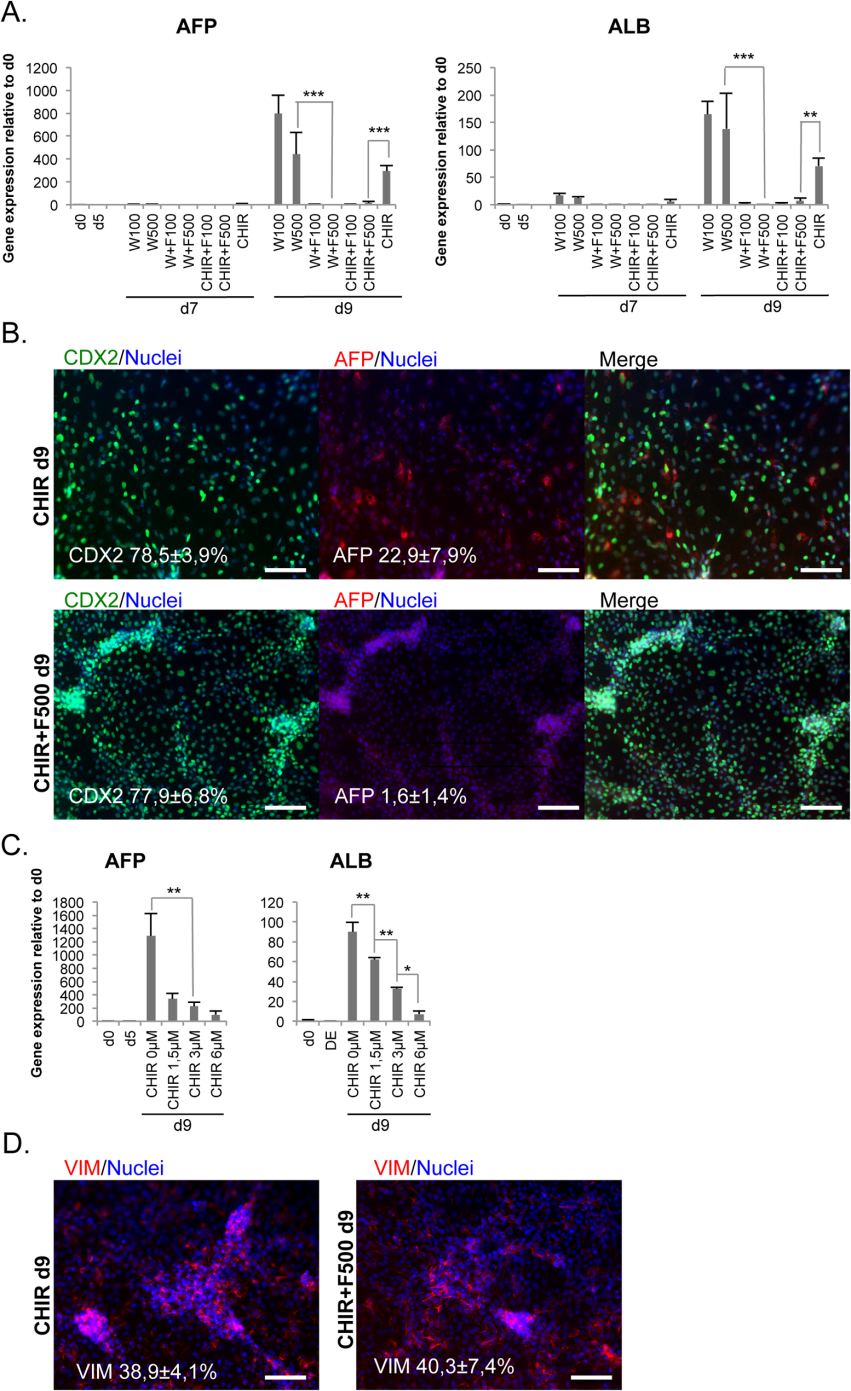
FGF4 suppresses unwanted hepatic differentiation. A. Expression of hepatic markers *AFP* (n = 4–15) and *ALB* (n = 4–14) during hindgut differentiation induced by different concentrations (ng/ml) of WNT3A (W) and FGF4 (F) and 3 μM CHIR99021 (mean ± SEM). B. Double immunocytochemistry for CDX2 and AFP at day 9. C. Expression levels of *AFP* and *ALB* during differentiation with varying concentrations of CHIR, (mean ± SEM; n = 3). D. Immunocytochemistry for VIM at day 9. Scale bars 100 μm.

We analyzed the expression of hepatic markers *AFP* and *ALBUMIN* at the hindgut stage to identify unwanted endodermal lineages (Fig 3A). These markers were clearly upregulated in conditions with WNT3A or CHIR alone. However, administration of FGF4 blunted the expression of hepatic markers (Fig 3A). Immunostaining for AFP confirmed the presence of positive cells at day 9 in WNT3A and CHIR conditions and their absence in the FGF4 treated cultures (Fig 3B). Increasing the concentration of CHIR also seemed to decrease *AFP* and *ALB* expression (Fig 3C), while *CDX2* expression was increased (Fig 2B). VIM positive cells were also detected in the day 9 cultures (Fig 3D).

Taken together, FGF4 was not necessary for the induction of *CDX2*, but it increased the specificity of the differentiation away from the hepatic lineage.

### Formation of organoids from hindgut cells is independent of R-spondin1

**Fig 4:**
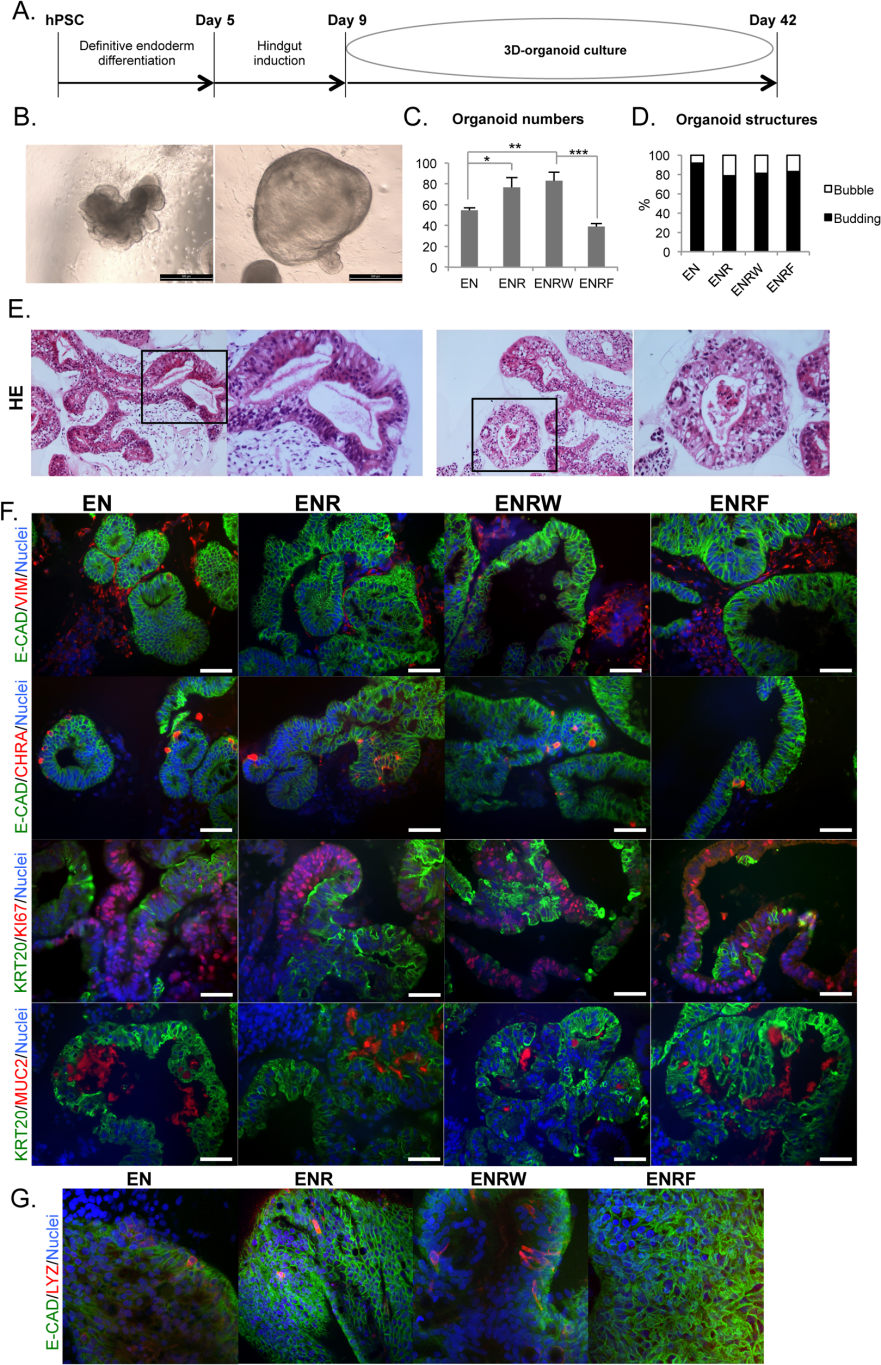
HPSC-derived hindgut cells form intestinal organoids in the absence of R-spondin1. Results represent day 33 organoids (d42 from hPSC) derived from day 9 CHIR cells. For CHIR+F cells see S4 Fig. A. Timeline representing the differentiation process B. Light microscopic images showing budding (left) vs. bubble-like (right) morphologies (scale bars 500 μm). C. Number of organoids in tested conditions (E, EGF; N, Noggin; R, R-Spondin1; W, WNT3A; F, FGF4). Error bars represent SD. D. Percentages of budding and bubble-like structures in the tested conditions. E. Hematoxylin-eosin (HE) stainings for organoid sections showing both tightly packed well-polarized structures (left panel) versus more loosely organized epithelium (righ panel). F. Immunohistochemistry for E-CADHERIN (E-CAD), VIMENTIN (VIM), CHROMOGRANIN A (CHRA), CYTOKERATIN 20 (KRT20), KI67 and MUCIN2 (MUC2) in organoids (scale bars 50 μm). G. Whole mount confocal immunocytochemistry for E-CAD and LYZ (magnification 40 x).

H9 cells at day 9 of differentiation were embedded in Matrigel for 3D-organoid culture (Fig 4A). Hereafter, the day 9 starting cell populations are referred as “CHIR” for cells induced to day 9 by CHIR alone, and “CHIR+F” for cells induced to day 9 by CHIR + FGF4 (500 ng/ml). During 3D-culture, organoids (S3 Fig) developed mostly from the condensed structures that started to form at the hindgut induction stage (S2 Fig). They later formed either budding, outpocketing-containing (>80%) or more uniform bubble-like (<20%) structures (Fig 4B). Four different culture conditions were tested for the organoids derived from CHIR cells: 1) Basal medium plus EGF+ Noggin (EN), 2) EGF + Noggin + R-spondin1 (ENR), 3) EGF +Noggin + R-spondin1 + WNT3A (ENRW) or 4) EGF + Noggin + R-spondin1 + FGF4 (ENRF). For the CHIR+F cells, only the EN and ENR conditions were tested.

**Fig 5:**
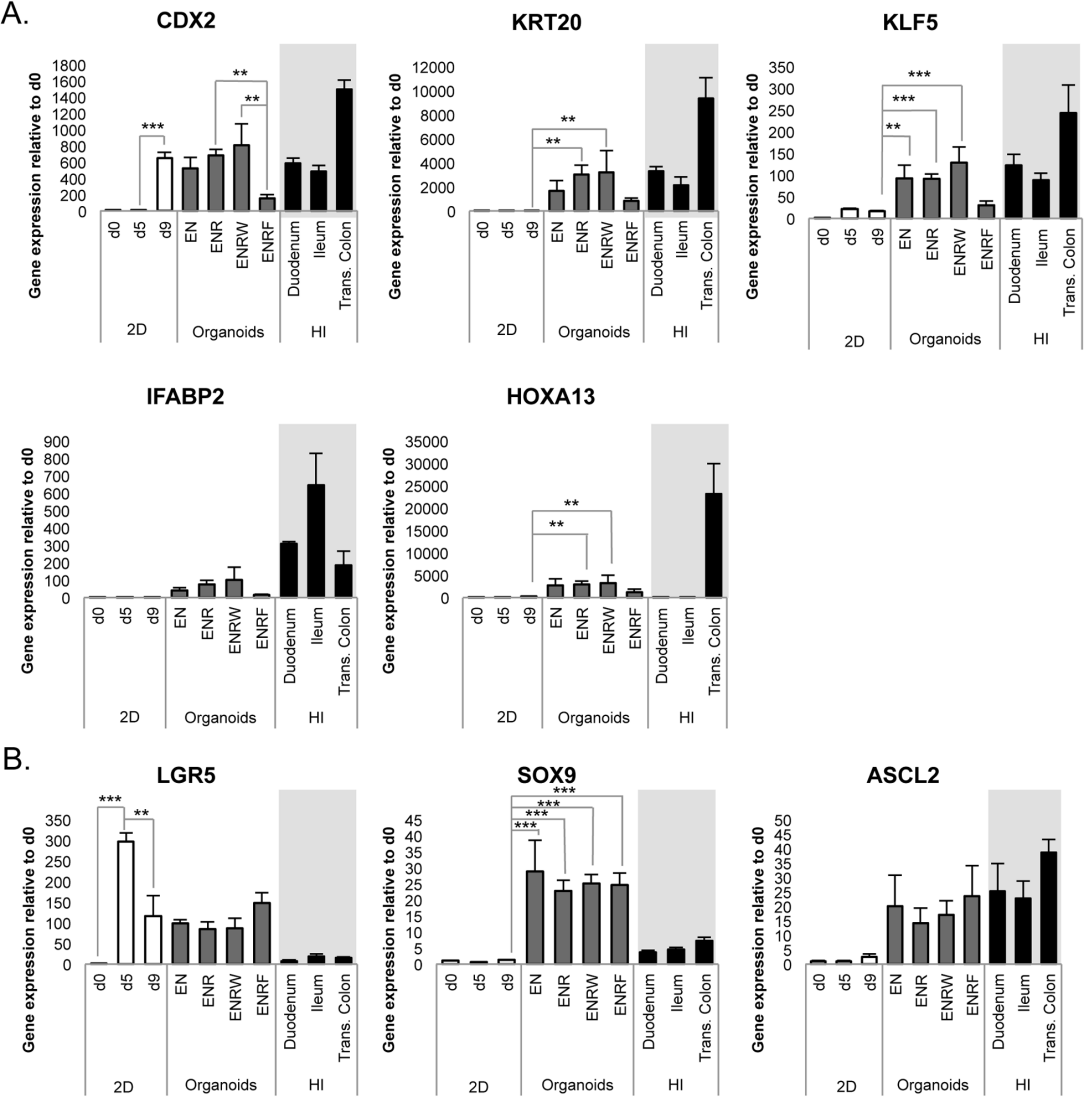
HPSC-derived organoids express intestinal marker genes at levels comparable to human intestinal epithelium. Results represent day 33 organoids (d42 from hPSC) derived from day 9 CHIR cells. A. Gene expression of intestinal differentiation markers *CDX2*, *KRT20*, *KLF5*, *IFABP2* and *HOXA13*. B. Expression of crypt area/intestinal stem cell markers *LGR5*, *ASCL2* and *SOX9*. Data represent the mean ± SEM of 3–9 samples. (E, EGF; N, Noggin; R, R-Spondin1; W, WNT3A; F, FGF4; 2D, initial monolayer culture; HI, human intestinal epithelium samples.)

Both the CHIR and CHIR+F cells formed organoids also in the EN-condition, in the absence of RSPO1 (Fig 4) (S3 and S4 Figs). FGF4 inhibited the formation of organoids from the CHIR cells, whereas the addition of RSPO1 and WNT3A increased it (Fig 4C). There were no evident differences in the proportions of budding vs. bubble-like morphologies between the tested conditions (Fig 4D). Hematoxylin-eosin stainings revealed that the organoids consisted of both well-polarized epithelium and epithelium with weaker organization (Fig 4E). No differences were evident between test conditions. All organoid cultures matured to contain intestinal-like cell types, as evidenced by KRT20, CHRA, MUC2 and LYZ positive cells (Fig 4F and 4G) (S4 Fig). The cultures contained both E-CADHERIN positive epithelium and VIM positive mesenchyme (Fig 4F) (S4 Fig) and the organoids presented proliferative versus differentiated regions, as evidenced by KRT20/KI67 staining (Fig 4F) (S4 Fig). The intestinal differentiation markers *CDX2*, *KRT20*, *KLF5* and *IFABP2* were expressed at levels comparable to human intestinal epithelium in the EN, ENR and ENRW conditions, but weakly in the ENRF-condition (Fig 5A).

The intestinal stem cell/crypt area markers *LGR5*, *ASCL2* and *SOX9* were expressed in all hPSC-derived organoid cultures (Fig 5B) (S5 Fig). Interestingly, LGR5 expression peaked already at the DE stage, and in the organoids its expression remained higher than in the intestinal biopsy samples. SOX9 expression was also elevated in the organoids as compared with the intestinal epithelium. However, *ASCL2* mRNA levels were comparable in the organoids and in the primary intestinal samples (Fig 5B).

Day 9 CHIR+F cells formed organoids similarly to CHIR cells, when tested in EN and ENR conditions (S3 Fig) No evident differences were found between cell compositions of organoids derived from these two different starting populations. (Fig 4 and Fig 5) (S4 and S5 Figs).

To validate the results with another cell line, the entire differentiation process was repeated with the human iPSC line HEL11.4. Hindgut differentiation was induced using CHIR and the organoids were cultured in the EN condition. (S6 Fig). There were no apparent differences between the results obtained with H9 and HEL11.4 cells. Thus, our results clearly show that, in contrast to the adult intestine-derived organoids, the Wnt-agonist R-Spondin1 is dispensable for the hPSC-derived organoids.

### Opposite effects of WNT3A and FGF4 on the cellular composition of the organoids

**Fig 6:**
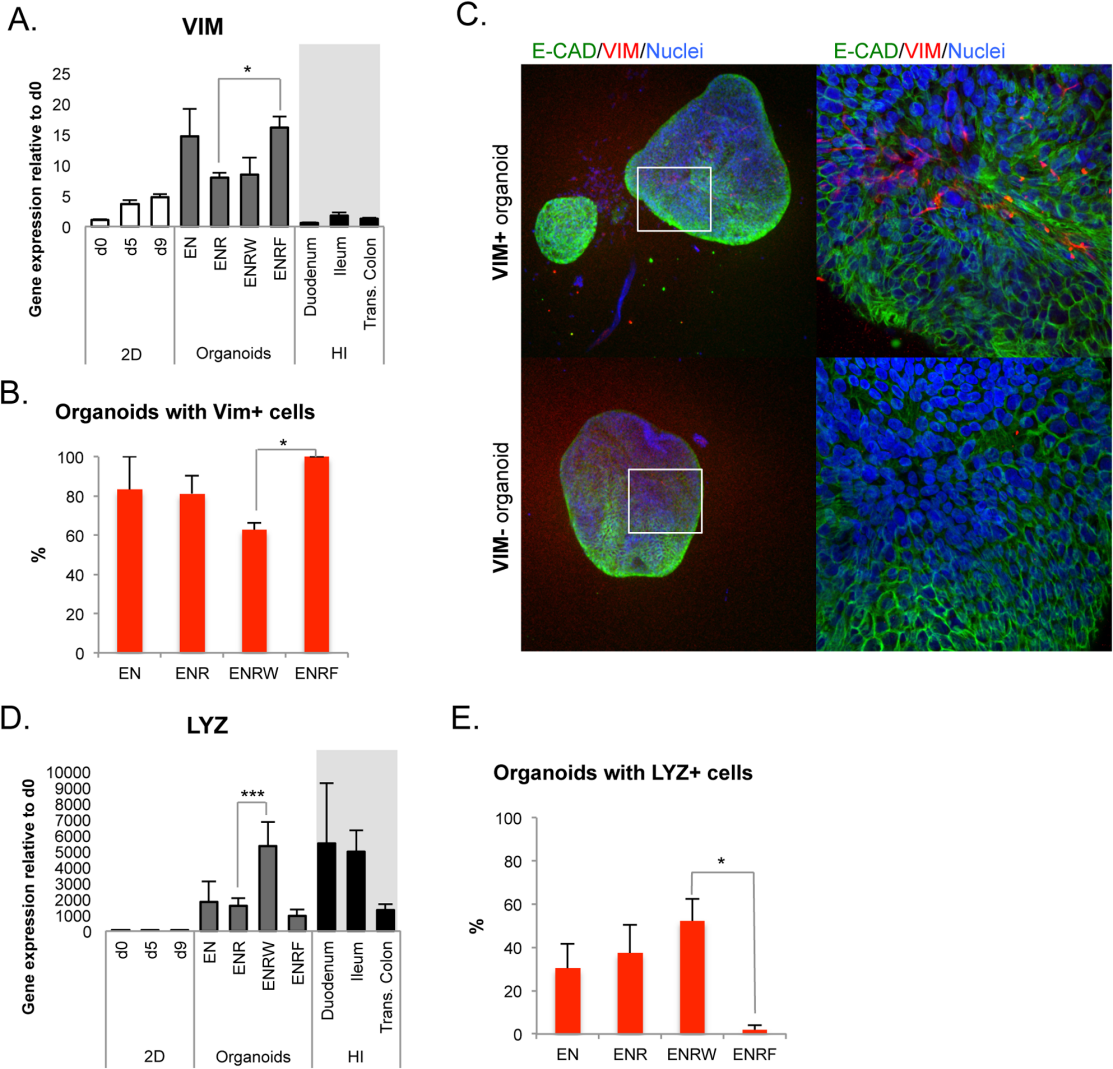
Opposite effects of WNT3A and FGF4 on mesenchymal and Paneth cells. A. QPCR analysis of *VIM* expression during the differentiation (mean ± SEM; n = 3–6). B. Percentages of organoids with VIM+ cells in the tested conditions (n = 2–3) C. Representative confocal Z-stack images of organoids with and without VIM+ cells. Left panel 10x, right panel 40x magnifications. D. QPCR analysis of *LYZ* expression during the differentiation (mean ± SEM; n = 3–8). E. Percentages of organoids with LYZ+ cells in the tested conditions (n = 3–6). All results represent day 33 organoids (d42 from hPSC) derived from day 9 CHIR cells.

Although the mesenchymal marker *VIM* was expressed in a subpopulation of cells at all stages and in all organoid culture conditions, *VIM* mRNA levels were significantly upregulated in the precence of FGF4 (ENRF condition, Fig 6A). When FGF4 was present, all the individual organoids examined contained VIM+ cells (Fig 6B and 6C). On the contrary, the proportion of VIM-positive organoids was lowest when the exogenous WNT3A ligand was present in the medium (ENRW condition, Fig 6B).

Lysozyme (LYZ) is a marker of the Paneth cells in the intestinal crypts. *LYZ* expression was elevated in the presence of exogenous WNT3A (ENRW, Fig 6D). However, based on immunocytochemistry, the presence of LYZ-containing organoids was not dependent on exogenous WNT, since quite similar percentages were found in EN, ENR and ENRW conditions (Fig 6E and Fig 4G). It is possible that WNT3A addition increased the number of Paneth cells in individual organoids where they had developed, which has been shown to occur in human adult intestinal organoids [22], but did not affect Paneth-cell negative organoids to the same extent. Interestingly, virtually no LYZ-positive cells were found in whole mount immunocytochemistry of organoids grown in the presence of FGF4 (ENRF, Fig 6E and Fig 4G).

Collectively, these results indicate that FGF4 stimulated and WNT3A inhibited the formation of mesenchyme within the organoids. An opposite effect was found for the Paneth cells, which were supported by WNT3A and virtually abolished by exogenous FGF4.

### WNT3A prolongs the survival of hPSC-derived intestinal organoids

All organoid cultures were initially analyzed after 33 days of 3D-Matrigel culture (d42 from hPSC). During this time, the cultures were split mechanically 3–4 times. In the EN, ENR and ENRW conditions, cells were cultured for up to 99 days (d108 from hPSC). In EN and ENR conditions, all organoid growth was arrested after approximately 70 days (S7A Fig). In the ENRW condition, structures (S7B and S7C Fig) contained E-CAD, VIM, KRT20, CDX2, CHRA and MUC2 positive cells (S7D Fig) still at d99. However, d99 immunohistochemistry revealed active CASPASE3 (CASP3) positivity also in the epithelial structures (S7D Fig), whereas at d33 CASP3+ cells were mostly localized in mesenchymal parts (S7E Fig). No differences between different test conditions were noticed. Although WNT3A addition prolonged organoid survival to some extent, other modifications to the culture conditions would be needed to improve their sustained growth and viability.

## Discussion

Relatively few studies have been published on the *in vitro* derivation of intestinal-like cells from hPSCs [13–17, 25]. Spence et al. (2011) used WNT3A and FGF4 to induce *CDX2* and hindgut development in definitive endoderm and achieved 3D organoid formation in the precence of R-spondin1 [17]. They showed that hPSC derived organoids have a mesenchymal compartment that is likely to originate at early stages of the differentiation process. Our results show that even more potent *CDX2* upregulation can be achieved with Wnt agonist CHIR, leaving FGF4 dispensable for intestinal differentiation. Also, the further formation of maturing 3D organoids is independent of exogenous Wnt agonists, like R-spondin1. Similarly to Spence et al., we identified a mesenchymal compartment within the cultures.

Many studies describe 3D-organoid cultures derived from isolated human and mouse intestinal epithelium, allowing sustainable expansion of intestinal stem cells *in vitro* [20, 23, 26, 27] These organoids (also called enteroids), consisting only of epithelial cells, fail to truly recapitulate the complex *in vivo* signaling network of the intestine, where the epithelial lining is in close contact with the mesenchyme, central nervous system and the vascular plexus. HPSC-derived intestinal organoids contain the mesenchymal component and have thus more potential to mimic various aspects of intestinal complexity [34]. In the present study, we show that mesenchymal *VIM* expressing cells are generated already during the first stage of differentiation, induction of definitive endoderm, where most of the cells acquire endodermal identity. The VIM+ stromal cells were found to be present at all later stages, potentially representing an important source of endogenous growth factors, including Wnts.

C*dx2* is considered to be a reliable marker for the intestinal lineage in the context of endodermal differentiation, since it becomes highly expressed in the early embryonic intestine [35]. It also antagonizes the foregut differentiation program and its conditional ablation in the mouse endoderm results in anterior homeotic transformation and gastric metaplasia [36, 37]. In our experiments, the highest upregulation of *CDX2* was obtained using the Wnt signaling agonist CHIR99021, outperforming high concentrations of the ligand WNT3A. CHIR also preserved high *AXIN2* levels that were achieved already during definitive endoderm induction, while WNT3A was less potent in activating this well-known Wnt target gene [38, 39] during intestinal commitment. Addition of a high concentration of FGF4 did not further potentiate the induction. However, FGF4 did efficiently repress the expression of hepatic genes *AFP* and *ALBUMIN* at the hindgut stage. Previously, FGF4 has been shown to inhibit early foregut development in chick embryos [2]. Our experiments are in agreement with this observation, showing that FGF4 effectively abrogated the differentiation of hPSCs to the hepatic lineage. This implies that FGF4 has some potential to increase the specificity of the lineage determination, even though our results show that it is dispensable in intestinal differentiation.

After the successful derivation of hindgut cells, we wanted to develop optimal conditions for their further propagation into self-renewing intestinal organoids. Based on previous studies, we decided that EGF and Noggin are essential components for this. Keeping these factors constant, we went on to study the roles of RSPO1, WNT3A and FGF4 in subsequent 3D-organoid cultures. Since we found out that FGF4 was not necessary for the intestinal commitment, we chose to use the d9 CHIR cells for the comparison of EN, ENR, ENRW and ENRF conditions. To validate that R-spondin1 was dispensable in organoid culture, we then tested EN and ENR conditions also with d9 CHIR+F cells. In all of the tested conditions, markers of intestinal cells became upregulated at the organoid stage, but to lesser extent upon FGF4 addition. Genes characteristic for absorptive enterocytes, such as *IFABP2* and *KRT20* [40] were expressed in the organoids at levels comparable to human intestinal epithelium. The continued expression of *CDX2* was expected because it is also expressed by adult enterocytes [41], while LYZ was indicative of Paneth cells and MUC2 of intestinal goblet cells.

*KLF5* [42, 43] and *SOX9* [44] are expressed in crypt areas in adult intestine and expression of *LGR5* [18] and *ASCL2* [45] is restricted to intestinal stem cells at the crypt base. *LGR5* was already highly upregulated at the DE stage of differentiation and its mRNA levels were higher in the organoids than in primary intestinal samples. We hypothesize that at the DE stage *LGR5* may be expressed in all endodermal cells, but in the organoids its expression becomes limited to a subpopulation of stem cells, possibly representing the ASCL2 positive intestinal stem cells, that are giving rise to the various other intestinal cell types. In accordance with this, *ASCL2* expression was only detected after the hindgut specification stage.

HOXA13 is the most posterior marker of the HOX genes, important in the regional specification of the intestine [46]. As expected, *HOXA13* was only found to be expressed in the human colonic epithelial samples and not in duodenum or ileum. The cultured organoids are likely to represent a mixture of anterior and posterior identities, since *HOXA13* was expressed in the organoids but at a low level compared to the colonic samples. Colonic crypts do not contain Paneth cells and thus LYZ is usually absent in colon. We found organoids with and without LYZ positive cells in EN, ENR and ENRW conditions. This also points to the possibility that the hPSC-derived organoids represent variable regional specificity, including both small intestinal and colonic fates. Interestingly, FGF4 inhibited the generation of LYZ-positive organoids. However, it is unlikely to be due to any posteriorizing effect of FGF4, since all the differentiation markers including *HOXA13* were also expressed at lower levels in this condition.

Overall, exogenous FGF4 appeared to inhibit the growth and maturation of intestinal epithelial structures. This is an apparent discrepancy with the observation that *VIM* expression was enhanced in organoids cultured in the presence of FGF4 (ENRF condition), which should reflect an increased mesenchymal compartment as a source of beneficial growth factors. There are several possible explanations to this. The direct negative effects of FGF4 on the epithelial cells may overrule the positive effect on mesenchyme. It is also likely that the stage-specific effects of mesenchymal signals vary depending on the added growth factors. A deeper analysis of the mesenchymal-epithelial interactions and the effects of FGF4 in this context would be needed to thoroughly address this issue.

Remarkably, all the intestinal-like cell types that were identified in organoids cultured in the presence of RSPO1 (ENR-condition) were present also in organoids that had been cultured without RSPO1 (EN-condition). This may indicate that the initial Wnt signaling activation until day 9 was sufficient to enable self-sufficient development and maturation of the hPSC-derived organoids. It is likely that both the mesenchymal cells and LYZ+ cells within the cultures secrete Wnt proteins or other Wnt agonists, since Wnt signaling is known to be essential for intestinal development and differentiation [3, 47–49]. In fact, the organoids cultured with both exogenous RSPO1 and WNT3A (ENRW condition) had less mesenchymal VIM+ cells and more LYZ+ cells. This is in line with previous observations, showing that Wnt from redundant sources supports the maintenance of intestinal epithelium and the level of Wnt signaling controls the number of Paneth cells in adult intestinal organoids [22]. Even modest changes in Wnt signaling activity can lead to significant increases or decreases in Paneth cell number without affecting the proliferation of crypt cells [50]. After one month in 3D-culture, the organoids displayed zones of proliferation versus differentiation, evidenced by KI67/KRT20 staining. However, the LYZ-positive cells did not clearly localize to crypt-like areas, indicating that mature crypts had not yet developed. At this stage apoptosis was not detected in the epithelial parts, but after propagation of the culture up to 3 months also epithelium stained positive for active CASP3.

In summary, our results provide insights into the process of intestinal development through the directed differentiation of hPSCs. We show that FGF4 is dispensable in intestinal differentiation *in vitro*, but has a minor role in repressing the hepatic lineage, while strong Wnt activation is essential and sufficient for the induction of the intestinal fate. We also show that *LGR5* is highly expressed already at the early definitive endoderm stage, limiting its reliability to serve as a marker unique of intestinal stem cells in hPSC-based applications. Furthermore, we successfully derived self-renewing 3D-organoids from hindgut cells that matured to contain all major intestinal cell types even without exogenous Wnt agonists. This highlights the remarkable level of self-organization achieved in epithelial-mesenchymal 3D cultures of stem cell derived committed progenitors. The iPSC-derived intestinal organoids provide a platform for modeling of a wide range of diseases, ranging from developmental defects to colon cancer.

## Supporting Information

S1 Fig Cell morphology and immunocytochemistry for SOX17/SOX2 and SOX17/VIM during definitive endoderm differentiation. Scale bars 100 μm.
(TIF)

S2 Fig Morphology of d9 (hindgut) cells differentiated with the tested protocols. Scale bars 500 μm. WNT3A (W), FGF4 (F) and CHIR99021 (CHIR). Numbers indicate the concentrations used in ng/ml. CHIR concentration was 3 μM.
(TIF)

S3 Fig Organoid morphology in different test conditions. Note that the mesenchymal cells are in close contact to the growing epithelial budding structures. Images were taken after 33 days in organoid culture (d42 from the start of the differentiation process.) Scale bars 100 μm or 500 μm. (E, EGF; N, Noggin; R, R-Spondin1; W, WNT3A; F, FGF4)
(TIF)

S4 Fig R-spondin1 does not affect the composition of organoids derived from CHIR+F treated day 9 cells. Immunofluorescent images of organoids cultured for 33 days without (EN) or with R-Spondin1 (ENR). Scale bars 50 μM.
(TIF)

S5 Fig Gene expression levels in organoids derived from CHIR+F treated d9 cells (qPCR). Experimental setup is similar to that presented in Fig 5.
(TIF)

S6 Fig Differentiation of the hiPSC cell line HEL11.4 to DE, hindgut (CHIR) and organoids (EN). A. Day 5 cells stained with OCT4/FOXA2. Scale bar 100 μm. B. CDX2 positive spheroids formed at day 9. Scale bar 200 μm. C. Representative histogram of flow cytometric analysis of the endodermal cell surface marker CXCR4 at day 5. D. Representarive histogram of flow cytometry for CDX2 at day 9. E. Representative light microscopic images of the organoids. F. qPCR analysis during the differentiation process (n = 1). G. Immunohistochemistry for organoid sections. Scale bars 50 μm.
(TIF)

S7 Fig hPSC-derived organoids have a limited life span in 3D culture. Survival of organoids in 3D-culture in EN, ENR and ENRW conditions (mean ± SEM; n = 2–5). (Data were combined from CHIR and CHIR+F derived organoids of H9 cells) B. Light microscope image of d99 organoids cultured in ENRW condition (d9 CHIR). Scale bar 500 μm. Notice the poor appearance compared to d33 organoids (S3 Fig). C. HE stainings for d99 organoids cultured in the ENRW condition. D. d99 ENRW organoids immunohistochemistry for E-CAD, VIM, CHRA, KRT20, KI67, MUC2, CDX2 and CASPASE3 (CASP3) E. d33 organoids immunohistochemistry for CASP3 showing that at this stage positive cells are mostly located in the non-epithelial parts in contrast to d99 (above). Scale bars 50 μm. (E, EGF; N, Noggin; R, R-Spondin1; W, WNT3A). d99 in 3D organoid culture = d108 from the start of the whole differentiation process.
(TIF)

S1 Table Primary antibodies. (DOCX)

S2 Table Secondary Antibodies—Alexa Fluor. (DOCX)

S3 Table Antibodies for flow cytometric analysis with CXCR4. (DOCX)

S4 Table Primers for qPCR. (DOCX)
