# Supplementary figures and images for: Intestinal Commitment and Maturation of Human Pluripotent Stem Cells Is Independent of Exogenous FGF4 and R-spondin1
Source: PLoS One. 2015 Jul 31;10(7):e0134551. doi: 10.1371/journal.pone.0134551 (PMC4521699; doi:10.1371/journal.pone.0134551)

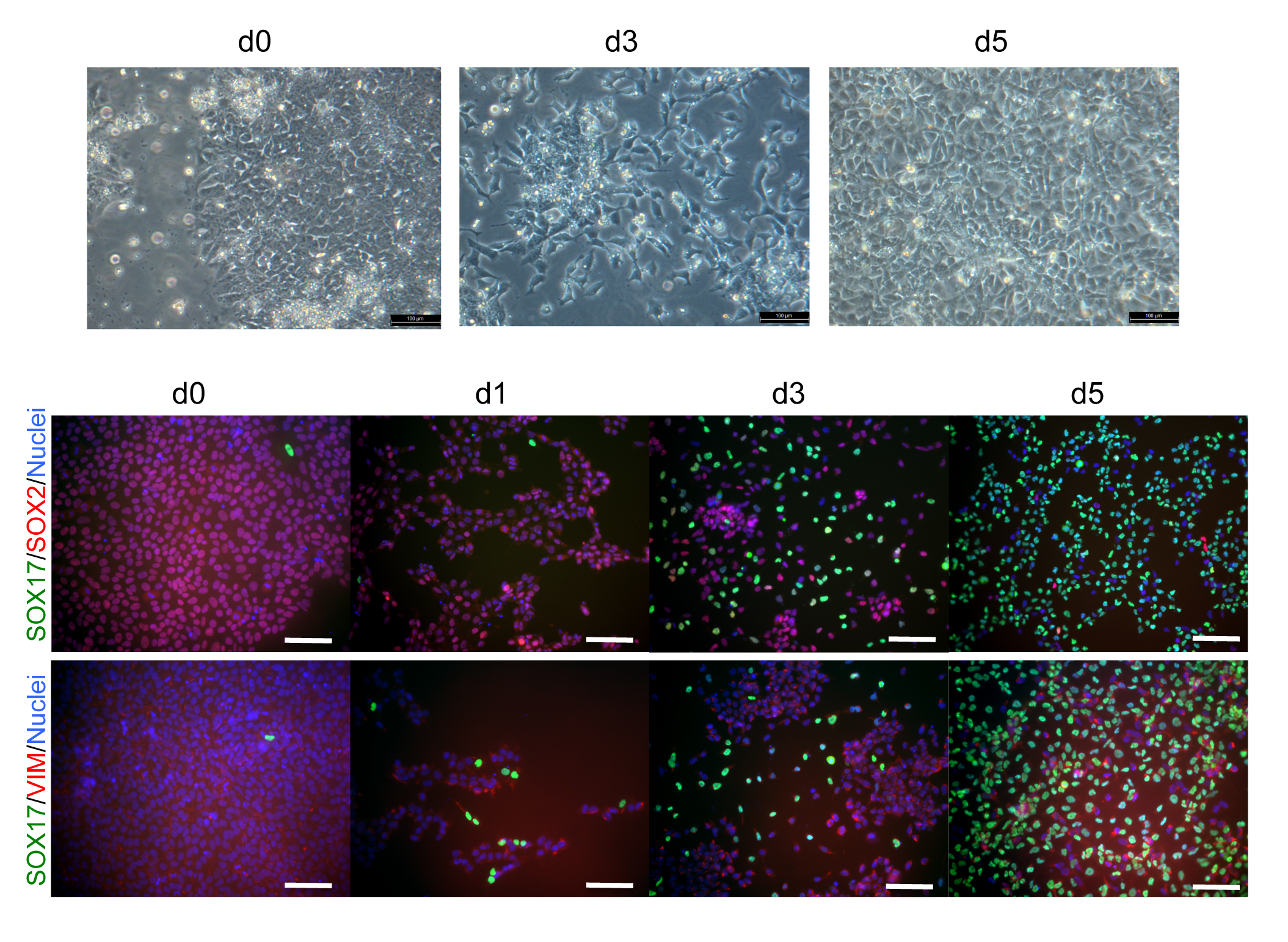

Supplement: S1 Fig — Scale bars 100 μm. (TIF) [file pone.0134551.s001.tif]

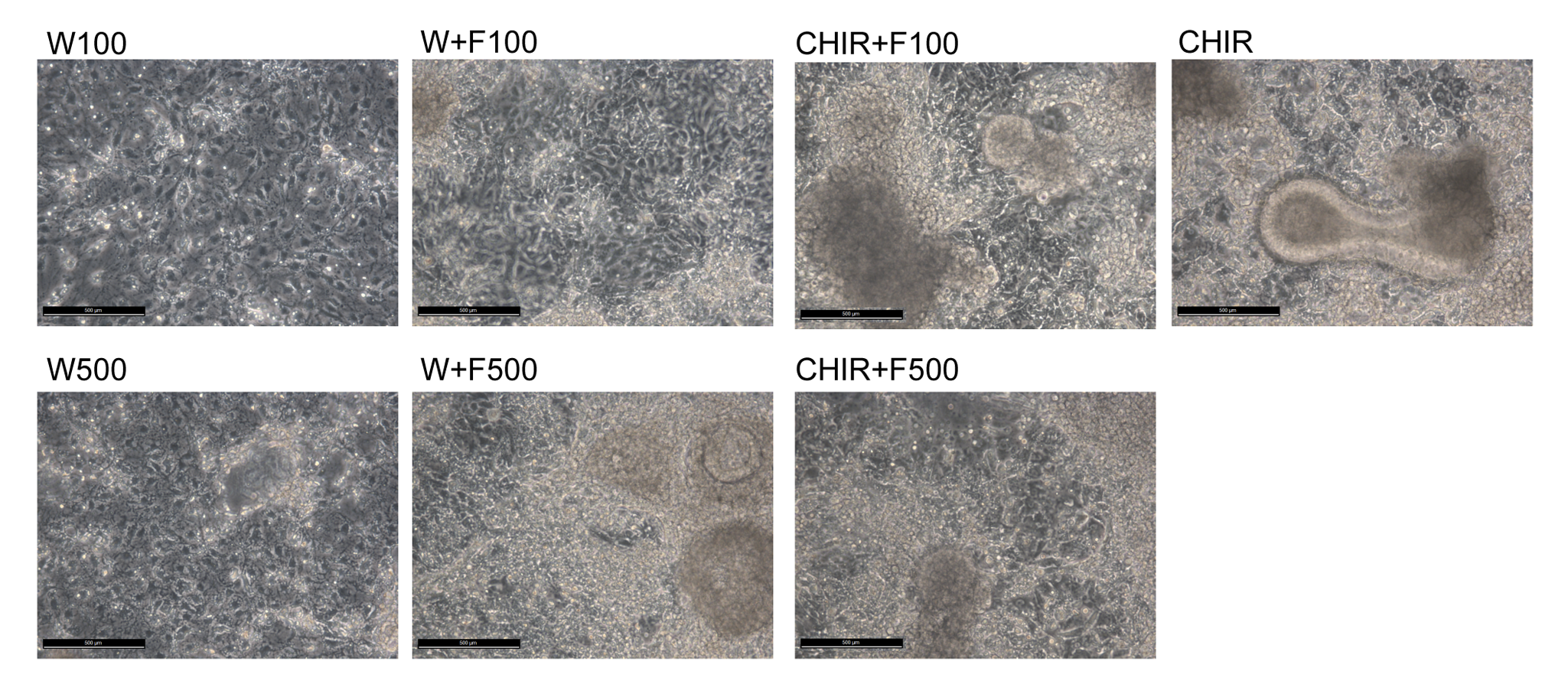

Supplement: S2 Fig — Scale bars 500 μm. WNT3A (W), FGF4 (F) and CHIR99021 (CHIR). Numbers indicate the concentrations used in ng/ml. CHIR concentration was 3 μM. (TIF) [file pone.0134551.s002.tif]

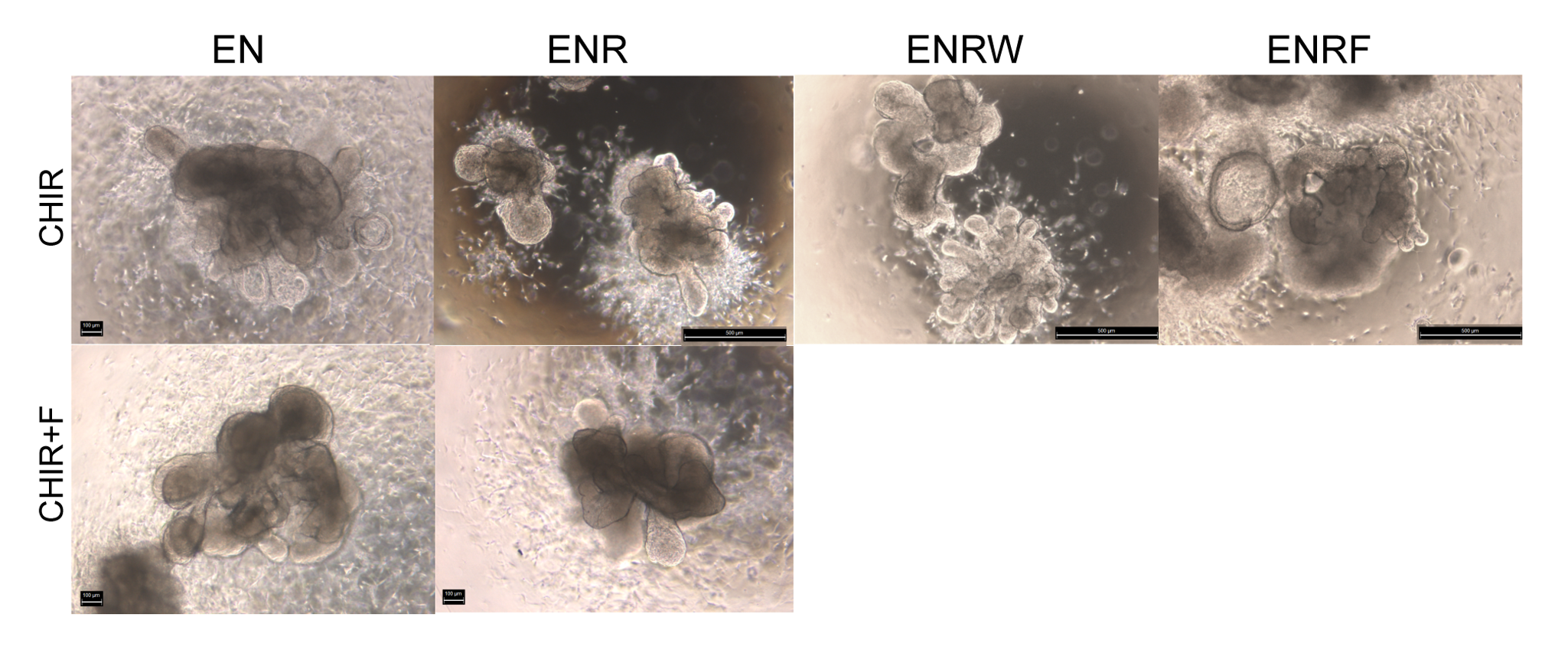

Supplement: S3 Fig — Note that the mesenchymal cells are in close contact to the growing epithelial budding structures. Images were taken after 33 days in organoid culture (d42 from the start of the differentiation process.) Scale bars 100 μm or 500 μm. (E, EGF; N, Noggin; R, R-Spondin1; W, WNT3A; F, FGF4) (TIF) [file pone.0134551.s003.tif]

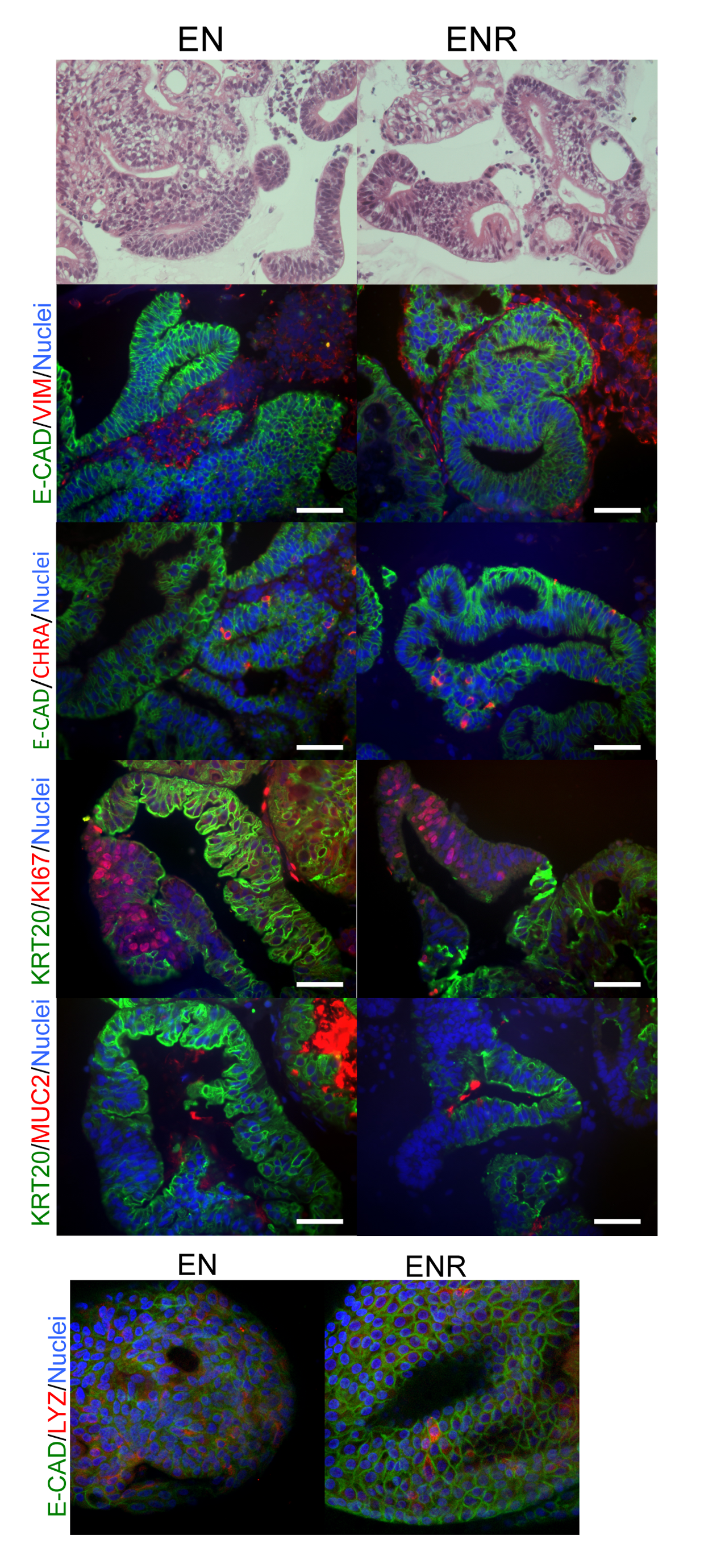

Supplement: S4 Fig — Immunofluorescent images of organoids cultured for 33 days without (EN) or with R-Spondin1 (ENR). Scale bars 50 μM. (TIF) [file pone.0134551.s004.tif]

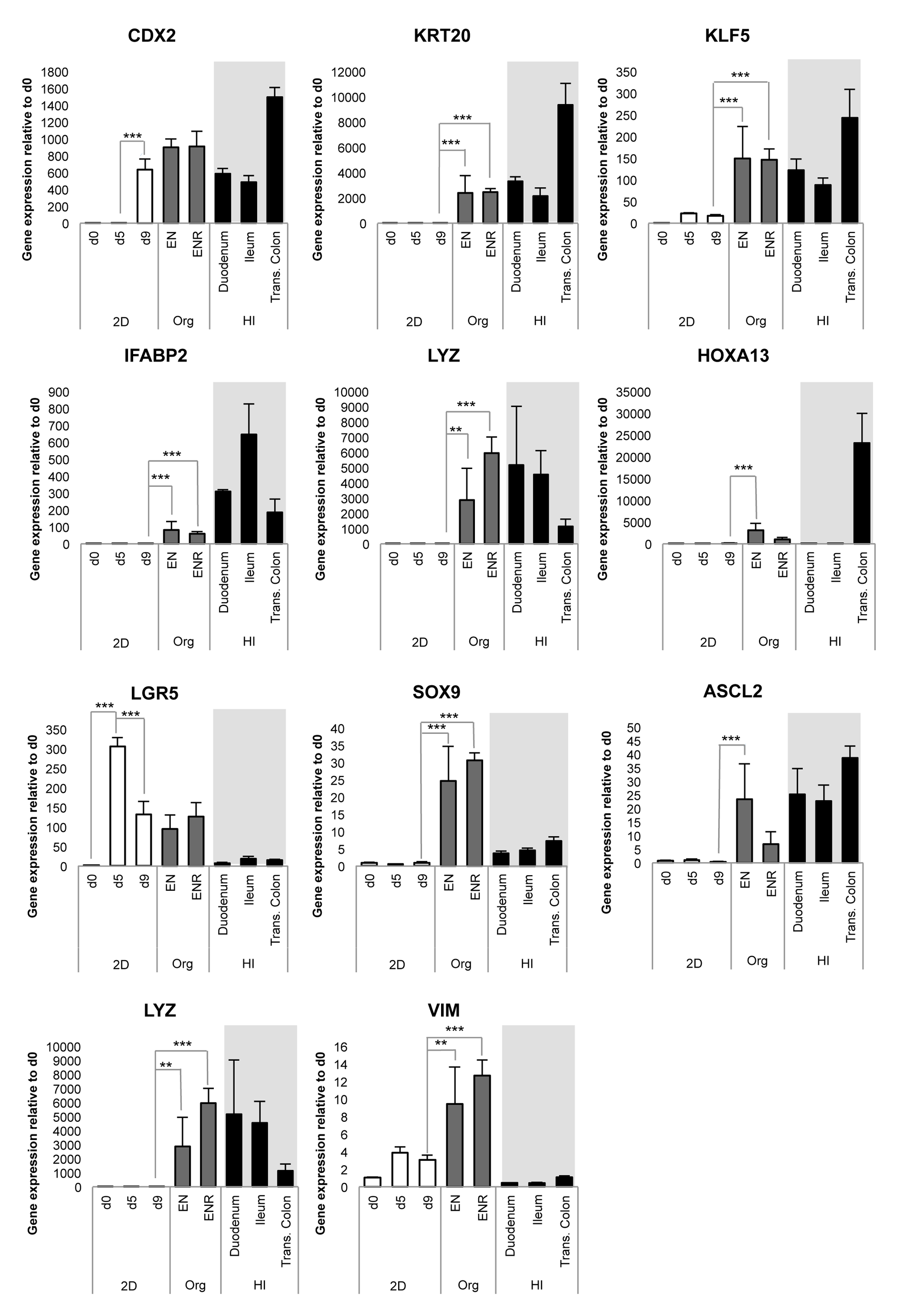

Supplement: S5 Fig — Experimental setup is similar to that presented in Fig 5. (TIF) [file pone.0134551.s005.tif]

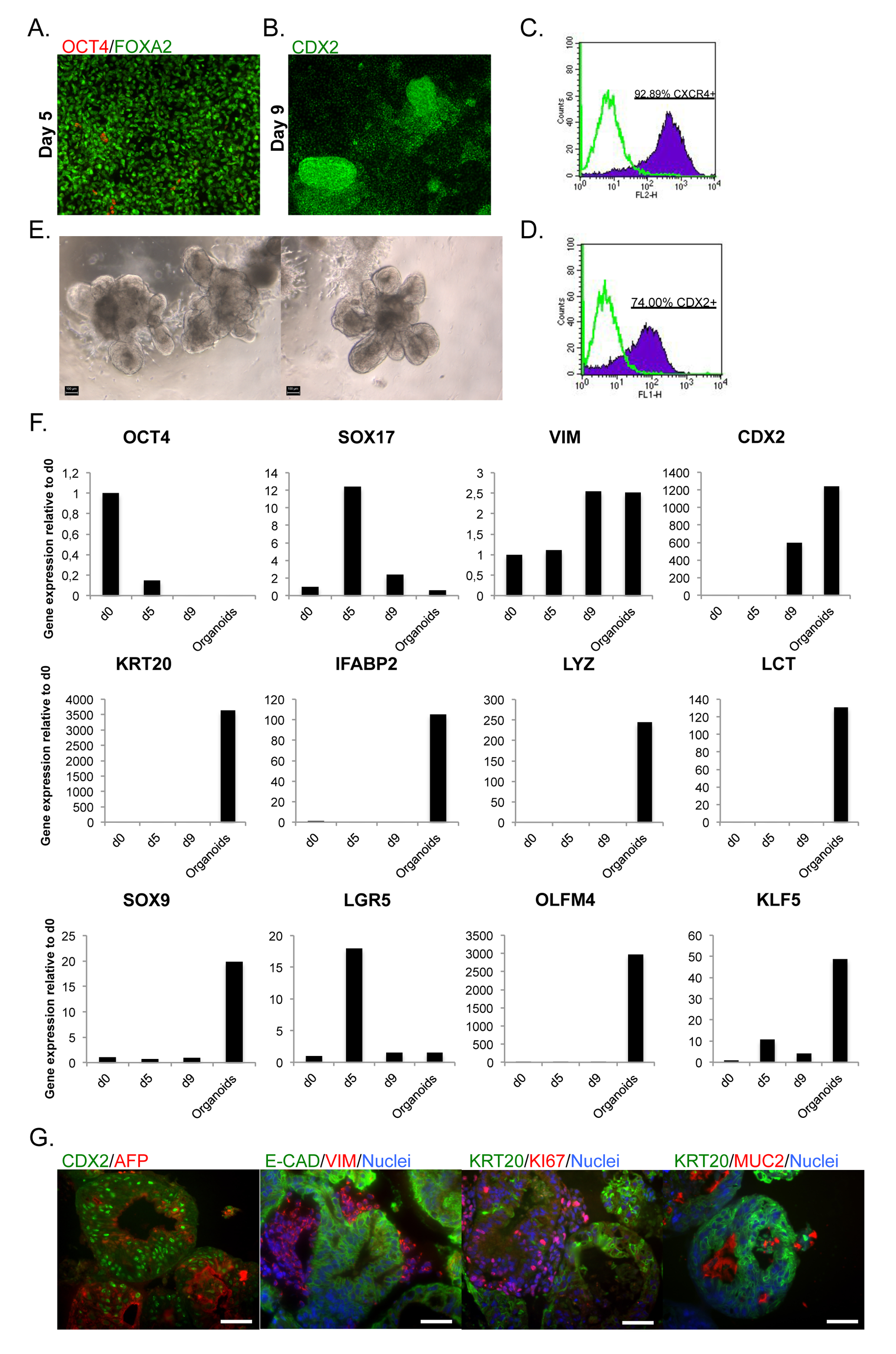

Supplement: S6 Fig — A. Day 5 cells stained with OCT4/FOXA2. Scale bar 100 μm. B. CDX2 positive spheroids formed at day 9. Scale bar 200 μm. C. Representative histogram of flow cytometric analysis of the endodermal cell surface marker CXCR4 at day 5. D. Representarive histogram of flow cytometry for CDX2 at day 9. E. Representative light microscopic images of the organoids. F. qPCR analysis during the differentiation process (n = 1). G. Immunohistochemistry for organoid sections. Scale bars 50 μm. (TIF) [file pone.0134551.s006.tif]

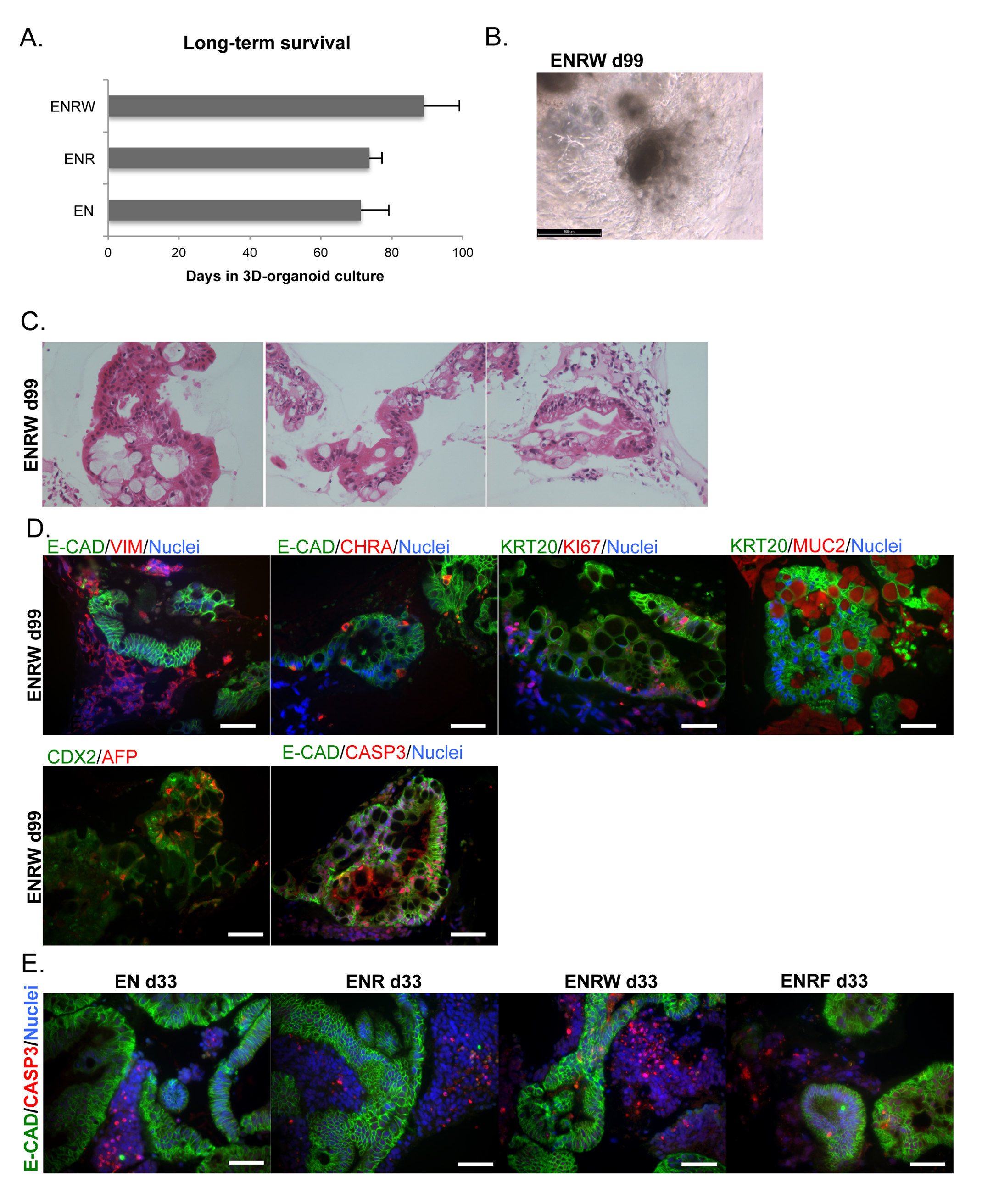

Supplement: S7 Fig — Survival of organoids in 3D-culture in EN, ENR and ENRW conditions (mean ± SEM; n = 2–5). (Data were combined from CHIR and CHIR+F derived organoids of H9 cells) B. Light microscope image of d99 organoids cultured in ENRW condition (d9 CHIR). Scale bar 500 μm. Notice the poor appearance compared to d33 organoids (S3 Fig). C. HE stainings for d99 organoids cultured in the ENRW condition. D. d99 ENRW organoids immunohistochemistry for E-CAD, VIM, CHRA, KRT20, KI67, MUC2, CDX2 and CASPASE3 (CASP3) E. d33 organoids immunohistochemistry for CASP3 showing that at this stage positive cells are mostly located in the non-epithelial parts in contrast to d99 (above). Scale bars 50 μm. (E, EGF; N, Noggin; R, R-Spondin1; W, WNT3A). d99 in 3D organoid culture = d108 from the start of the whole differentiation process. (TIF) [file pone.0134551.s007.tif]
