# Supplementary material for: Intestinal Commitment and Maturation of Human Pluripotent Stem Cells Is Independent of Exogenous FGF4 and R-spondin1
Source: PLoS One. 2015 Jul 31;10(7):e0134551. doi: 10.1371/journal.pone.0134551 (PMC4521699; doi:10.1371/journal.pone.0134551)
Supplement: S1 Table — (DOCX) [file pone.0134551.s008.docx]

**Table S1. Primary antibodies**

| **Antibody** | **Manufacturer** | **Catalog Number** | **Description** | **Dilution** |
| --- | --- | --- | --- | --- |
| CDX2 | BioGenex | MU392A-UC | mouse monoclonal IgG | 1:500 |
| FOXA2 / HNF3beta (P-19) | Santa Cruz Biotechnology | sc-9187 | goat polyclonal IgG | 1:500 |
| OCT3/4 (H-134) | Santa Cruz Biotechnology | sc-9081 | rabbit polyclonal IgG | 1:500 |
| SOX2 | Cell Signaling | D6D9 | Rabbit monoclonal IgG | 1:500 |
| SOX17 | R&D Systems | AF1924 | Polyglonal goat IgG | 1:500 |
| CHRA | Santa Cruz Biotechnology | sc-13090 | rabbit polyclonal IgG | 1:100 |
| MUCIN2 | Santa Cruz Biotechnology | sc-15334 | rabbit polyclonal IgG | 1:200 |
| E-CADHERIN | BD transduction laboratories | 610181 | mouse | 1:250 |
| KRT20 | Dako | M7019 | mouse | 1:500 |
| VIM | Santa Cruz Biotechnology | sc-5565 | rabbit polyclonal IgG | 1:500 |
| KI67 | Leica | NCL-Ki67p | rabbit polyclonal IgG | 1:500 |
| AFP | Dako | A0008 | rabbit polyclonal | 1:500 |
| LYZ | Dako | A 0099 | Rabbit | 1:200 |
| CASP3 (active) | R&D Systemms | AF835 | Rabbit | 1:400 |
