# Supplementary material for: Intestinal Commitment and Maturation of Human Pluripotent Stem Cells Is Independent of Exogenous FGF4 and R-spondin1
Source: PLoS One. 2015 Jul 31;10(7):e0134551. doi: 10.1371/journal.pone.0134551 (PMC4521699; doi:10.1371/journal.pone.0134551)
Supplement: S2 Table — (DOCX) [file pone.0134551.s009.docx]

**Table S2. Secondary Antibodies - Alexa Fluor®**

| A11055 | Donkey Anti-goat IgG | Alexa Fluor® 488 | 1:500 |
| --- | --- | --- | --- |
| A11058 | Donkey Anti-goat IgG | Alexa Fluor® 594 | 1:500 |
| A21202 | Donkey Anti-mouse IgG | Alexa Fluor® 488 | 1:500 |
| A21203 | Donkey Anti-mouse IgG | Alexa Fluor® 594 | 1:500 |
| A21206 | Donkey Anti-rabbit IgG | Alexa Fluor® 488 | 1:500 |
| A21207 | Donkey Anti-rabbit IgG | Alexa Fluor® 594 | 1:500 |
| A21447 | Donkey Anti-goat IgG | Alexa Fluor® 647 | 1:500 |
