# Supplementary material for: Intestinal Commitment and Maturation of Human Pluripotent Stem Cells Is Independent of Exogenous FGF4 and R-spondin1
Source: PLoS One. 2015 Jul 31;10(7):e0134551. doi: 10.1371/journal.pone.0134551 (PMC4521699; doi:10.1371/journal.pone.0134551)
Supplement: S3 Table — (DOCX) [file pone.0134551.s010.docx]

**Table S3. Antibodies for flow cytometric analysis with CXCR4**

| **Name** | **Manufacturer** | **Catalog number** | **Lot number** |
| --- | --- | --- | --- |
| PE Mouse Anti-Human CD184 (CXCR4) | BD Bioscience | Cat:555974 | Lot:54058 |
| PE Mouse IgG2a, K Isotype Control | BD Bioscience | Cat:555574 | Lot:84599 |
