# Supplementary material for: Intestinal Commitment and Maturation of Human Pluripotent Stem Cells Is Independent of Exogenous FGF4 and R-spondin1
Source: PLoS One. 2015 Jul 31;10(7):e0134551. doi: 10.1371/journal.pone.0134551 (PMC4521699; doi:10.1371/journal.pone.0134551)
Supplement: S4 Table — (DOCX) [file pone.0134551.s011.docx]

**Table S4. Primers for qPCR**

| **Primer name** | **Origin** | **Sequence of 5'-primer (F)** | **Sequence of 3'-primer (R)** | **Product size** |
| --- | --- | --- | --- | --- |
| *ALB* | hum (DQ986150) | GGAAAAGTGGGCAGCAAATGT | GGTTCAGGACCACGGATAGA | 85 bp |
| *AFP* | hum (NM_001134.1) | CGCTGCAAACGATGAAGCAAG | AATCTGCAATGACAGCCTCAAG | 92 bp |
| AXIN2 | hum (NM_004655.3) | TCAGAGCGATGGATTTCGGGG | CTCTCAAGTCAGCAGGGGCT | 101 bp |
| *CDX1* | hum (NM_001804 ) | GACGCCCTACGAGTGGATG | TGTAGACCACGCGGTACTTG | 95 bp |
| *CDX2* | hum (NM_001265) | CCAGCGGCGGAACCTGTG | GTCTTTCGTCCTGGTTTTCAC | 82 bp |
| *CYCLOG* | hum (NM_004792) | TCTTGTCAATGGCCAACAGAG | GCCCATCTAAATGAGGAGTTG | 84 bp |
| *KLF5* | hum (NM_001730.3) | CATCCACTACTGCGATTACCC | CCCAGGTACACTTGTATGGC | 110 bp |
| *LGR5* | hum (NM_003667.2) | GGAAATCATGCCTTACAGAGC | CACTCCAAATGCACAGCACTG | 99 bp |
| *OCT4* | hum (NM_002701) | TTGGGCTCGAGAAGGATGTG | TCCTCTCGTTGTGCATAGTCG | 91 bp |
| *HOXA13* | hum (NM_000522.4) | TGAACGGGAATACGCCACGA | GACCTGCCGCTCAGAGAGAT | 88 b |
| *LCT* | hum (NM_002299.2) | TGCCTTCCTGGACTATGCGG | GTAGCTCATCACCCACGGCT | 94 bp |
| *SOX17* | hum (NM_022454) | CCGAGTTGAGCAAGATGCTG | TGCATGTGCTGCACGCGCA | 103 bp |
| *IFABP2* | hum (NM_000134.3) | CGCCCAAGGACAGACCTGAAT | TTCCAAGTGCTGTCAAACGCC | 78 bp |
| *KRT20* | hum (NM_019010.2) | ACTAACGGAGCTGAGACGCA | GTAACGGGCCTTGGTCTCCT | 109 bp |
| *LYZ* | Hum (NM_000239.2) | CCCTGGTCAGCCTAGCACTC | CCTTGCCCTGGACCGTAACA | 94 bp |
| *ASCL2* | hum (NM_005170) | GCGTTCCGCCTACTCGTCG | CTCAGTAGCCCCCTAACCAG | 101 bp |
| *OLFM4* | hum (NM_006418.4) | TGCCATTCGCCGAGAAATCG | GGTGGACGACAGGGGTGTTT | 84 bp |
| *SOX9* | hum (NM_000346) | ATCAAGACGGAGCAGCTGAG | GGCTGTAGTGTGGGAGGTTG | 100 bp |
| *VIM* | (NM_003380) | GGATTCACTCCCTCTGGTTG | TCGTGATGCTGAGAAGTTTCG | 105 bp |
